# SHEA/IDSA/APIC Practice Recommendation: Strategies to prevent healthcare-associated infections through hand hygiene: 2022 Update

**DOI:** 10.1017/ice.2022.304

**Published:** 2023-03

**Authors:** Janet B. Glowicz, Emily Landon, Emily E. Sickbert-Bennett, Allison E. Aiello, Karen deKay, Karen K. Hoffmann, Lisa Maragakis, Russell N. Olmsted, Philip M. Polgreen, Polly A. Trexler, Margaret A. VanAmringe, Amber R. Wood, Deborah Yokoe, Katherine D. Ellingson

**Affiliations:** 1 Division of Healthcare Quality and Promotion, Centers for Disease Control and Prevention, Atlanta, Georgia; 2 Department of Infectious Diseases, MacLean Center for Clinical Medical Ethics, The University of Chicago Medical Center, Chicago, Illinois; 3 Department of Infectious Diseases and Department of Epidemiology, University of North Carolina Chapel Hill, North Carolina; 4 Carolina Antimicrobial Stewardship Program, UNC Gillings School of Global Public Health, UNC Hospitals, Chapel Hill, North Carolina; 5 Department of Epidemiology, Robert N. Butler Columbia Aging Center, Columbia University, New York, New York; 6 Association of periOperative Registered Nurses (AORN), Denver, Colorado; 7 Division of Infectious Diseases, University of North Carolina School of Medicine, Chapel Hill, North Carolina; 8 Department of Medicine, Johns Hopkins University School of Medicine, The Johns Hopkins Hospital, Baltimore, Maryland; 9 Trinity Health, Livonia, Michigan; 10 Department of Internal Medicine, Carver College of Medicine, University of Iowa, Iowa City, Iowa; 11 Healthcare Epidemiology and Infection Control, The Johns Hopkins Health System, Baltimore, Maryland; 12 The Joint Commission, Office of Public Policy and Government Relations, Washington, DC; 13 Department of Medicine, University of California San Francisco, UCSF Health–UCSF Medical Center, San Francisco, California; 14 Department of Epidemiology and Biostatistics, College of Public Health, The University of Arizona, Tucson, Arizona

## Abstract

The purpose of this document is to highlight practical recommendations to assist acute-care hospitals in prioritization and implementation of strategies to prevent healthcare-associated infections through hand hygiene. This document updates the *Strategies to Prevent Healthcare-Associated Infections in Acute Care Hospitals through Hand Hygiene*, published in 2014. This expert guidance document is sponsored by the Society for Healthcare Epidemiology (SHEA). It is the product of a collaborative effort led by SHEA, the Infectious Diseases Society of America, the Association for Professionals in Infection Control and Epidemiology, the American Hospital Association, and The Joint Commission, with major contributions from representatives of a number of organizations and societies with content expertise.

## Purpose

Previously published guidelines provided comprehensive recommendations for detecting and preventing healthcare-associated infections (HAIs). The intent of this document is to highlight practical recommendations in a concise format designed to assist acute-care hospitals in implementing and prioritizing efforts to prevent HAIs through hand hygiene. This document updates the *Strategies to Prevent Healthcare-Associated Infections through Hand Hygiene*, published in 2014. This expert guidance document is sponsored by the SHEA. It is the product of a collaborative effort led by the SHEA, the Infectious Diseases Society of America (IDSA), the Association for Professionals in Infection Control and Epidemiology (APIC), the American Hospital Association (AHA), and The Joint Commission, with major contributions from representatives of organizations and societies with content expertise.

## Summary of major changes

This section lists major changes from the *Strategies to Prevent Healthcare-Associated Infections Through Hand Hygiene: 2014 Update*, including recommendations that have been added, removed, or altered. Recommendations in this document are categorized as “essential practices” that are foundational to all HAI programs in acute-care hospitals. In 2014, these were “basic practices,” renamed to highlight their importance as foundational for hospitals’ healthcare-associated infection (HAI) prevention programs. Some recommendations are “additional approaches” that may be considered for use in locations and/or populations within hospitals during outbreaks or when HAIs are not controlled after implementation of essential practices. In 2014, these were “special approaches.” A complete summary of the recommendations contained in this document is provided in Table 1.

**Table 1.** Summary of Recommendations to Prevent Healthcare-Associated Infections through Hand Hygiene

Essential Practices		
1.	**Promote the maintenance of healthy hand skin and fingernails.** ^10,57,58,154^ (Quality of Evidence: HIGH)	
	a.	Promote the preferential use of ABHS in most clinical situations^10,64^ (Quality of evidence: HIGH)
	b.	Perform hand hygiene as indicated by the CDC or the WHO Five Moments. (Quality of evidence: HIGH)
	c.	Include fingernail care in facility-specific policies related to hand hygiene.^56,152^ (Quality of evidence: HIGH) • HCP should maintain short, natural fingernails. • Nails should not extend past the fingertip. • HCP who provide direct or indirect care in high-risk areas (eg, ICU, perioperative) should not wear artificial fingernail extenders. • Prohibitions against fingernail polish (standard or gel shellac) are at the discretion of the infection prevention program, except among scrubbed individuals who interact with the sterile field during surgical procedures; these individuals should not wear fingernail polish or gel shellac.
	d.	Include measures for primary and secondary prevention of dermatitis.
	e.	Provide HCP with readily accessible, facility-approved hand moisturizers.^64^
	f.	Engage all HCP in primary prevention of occupational irritant and allergic contact dermatitis.^62–64,154,155^ (Quality of evidence: HIGH)
	g.	Provide cotton glove liners for HCP with hand irritation and educate these HCP on their use.^64^ (Quality of evidence: MODERATE)
2.	**Select appropriate products.**	
	a.	For routine hand hygiene, choose liquid, gel, or foam ABHS with at least 60 % alcohol.^10,65,76,78,79^ (Quality of evidence: HIGH)
	b.	Involve HCP in selection of products.^147^ (Quality of evidence: HIGH)
	c.	Obtain and consider manufacturers’ product-specific data if seeking ABHS with ingredients that may enhance efficacy against organisms anticipated to be less susceptible to biocides.^78,79^ (Quality of evidence: MODERATE)
	d.	Confirm that the volume of ABHS dispensed is consistent with the volume shown to be efficacious.^89,95,98^ (Quality of evidence: HIGH)
	e.	Educate HCP about an appropriate volume of ABHS and the time required to obtain effectiveness.^95^ (Quality of evidence: HIGH)
	f.	Provide facility-approved hand moisturizer that is compatible with antiseptics and gloves. (Quality of evidence: HIGH)
	g.	For surgical antisepsis, use an FDA-approved surgical hand scrub or waterless surgical hand rub. (Quality of evidence: HIGH)
3.	**Ensure the accessibility of hand hygiene supplies**. (Quality of evidence: HIGH)	
	a.	Ensure that ABHS dispensers are unambiguous, visible, and accessible within the workflow of HCP.^105–111^ (Quality of evidence: HIGH)
	b.	In private rooms, consider 2 ABHS dispensers the minimum threshold for adequate numbers of dispensers: 1 dispenser in the hallway, and 1 in the patient room.^102^ (Quality of evidence: HIGH)
	c.	In semiprivate rooms, suites, bays, and other multipatient bed configurations, consider 1 dispenser per 2 beds the minimum threshold for adequate numbers of dispensers. Place ABHS dispensers in the workflow of HCP.^48^ (Quality of evidence: LOW)
	d.	Ensure that the placement of hand hygiene supplies (eg, individual pocket-sized dispensers, bed-mounted ABHS dispenser, single-use pump bottles) is easily accessible for HCP in all areas where patients receive care.^103,104^ (Quality of evidence: HIGH)
	e.	Evaluate the risk of intentional consumption. Utilize dispensers that mitigate this risk, such as wall-mounted dispensers that allow limited numbers of activations within short periods (eg, 5 seconds). (Quality of evidence: LOW)
	f.	Have surgical hand rub and scrub available in perioperative areas. (Quality of evidence: HIGH)
	g.	Consider providing ABHS hand rubs or handwash with FDA-approved antiseptics for use in procedural areas and prior to high-risk bedside procedures (eg, central-line insertion). (Quality of evidence: LOW)
4.	**Ensure appropriate glove use to reduce hand and environmental contamination.** ^130–132,138^ (Quality of evidence: HIGH)	
	a.	Use gloves for all contact with the patient and environment as indicated by standard and contact precautions during care of individuals with organisms confirmed to be less susceptible to biocides (eg, *C. difficile*, norovirus).^10^
	b.	Educate HCP about the potential for self-contamination and environmental contamination when gloves are worn. (Quality of evidence: HIGH)
	c.	Clean hands immediately following glove removal. If handwashing is indicated and sinks are not immediately available, use ABHS and then wash hands as soon as possible.
	d.	Educate and confirm the ability of HCP to doff gloves in a manner that avoids contamination. (Quality of evidence: HIGH)
5.	**Take steps to reduce environmental contamination associated with sinks and sink drains.** ^114,116–123^ (Quality of evidence: HIGH)	
	a.	Ensure that handwashing sinks are constructed according to local administrative codes.
	b.	Include handwashing sinks in water infection control risk assessments for healthcare settings.
	c.	If possible, dedicate sinks to handwashing.
	d.	Educate HCP to refrain from disposing substances that promote growth of biofilms (eg, intravenous solutions, medications, food, or human waste) in handwashing sinks.
	e.	Use an EPA-registered hospital disinfectant to clean sink bowls and faucets daily.
	f.	Do not keep medications or patient care supplies on countertops or mobile surfaces that are within 1m (3 feet) of sinks.
	g.	Provide disposable or single-use towels to dry hands. Do not use hot air dryers in patient care areas.
	h.	Consult with state or local public health officials when investigating confirmed or suspected outbreaks of healthcare-associated infections due to waterborne pathogens of plumbing in the facility.
6.	**Monitor adherence to hand hygiene.** (Quality of evidence: HIGH)	
	a.	Use multiple methods to measure adherence to hand hygiene.
	b.	Consider advantages and limitations of each type of monitoring.
7.	**Provide timely and meaningful feedback to enhance a culture of safety.** ^50–52^ (Quality of evidence: MODERATE)	
	a.	Provide feedback in multiple formats (eg, verbal, written) and on multiple occasions (eg, real-time, weekly).^50^
	b.	Consider debriefing unit managers as soon as possible after each direct covert observation session. This can be conducted in a manner that preserves the observer’s confidentiality.
	c.	Provide meaningful data with clear targets linked to actions that improve adherence.^50^
Additional Approaches during Outbreaks		
1.	Consider educating HCP using a structured approach (eg, WHO Steps) for handwashing or hand sanitizing. Evaluate HCP adherence to technique. (Quality of evidence: LOW)	
2.	For waterborne pathogens of premise plumbing, consider disinfection of sink drains using an EPA-registered disinfectant with claims against biofilms. Consult with state or local public health for assistance in determining appropriate protocols for use and other actions needed to ensure safe supply. (Quality of evidence: LOW)	
3.	For *Clostridioides difficile* and norovirus, in addition to contact precautions, encourage hand washing with soap and water after the care of patients with known or suspected infections. (Quality of evidence: LOW)	
Approaches that Should Not Be Considered a Routine Part of Hand Hygiene		
1.	Do not supply individual pocket-sized ABHS dispensers in lieu of minimum thresholds for accessible wall-mounted dispensers.	
2.	Do not refill or “top-off” soap dispensers, moisturizer dispensers, or ABHS dispensers intended for single use.^127^	
3.	Do not use antimicrobial soaps formulated with triclosan as an active ingredient.^67^	
4.	Do not routinely double-glove except when specifically recommended for certain job roles or in response to certain high-consequence pathogens.^141^	
5.	Do not routinely disinfect gloves during care except when specifically recommended in response to certain high-consequence pathogens.	
6.	Do not remove access to ABHS when responding to organisms that are anticipated to be less susceptible to biocides (eg, *C. difficile* or norovirus).^11^	
7.	Do not attempt to remediate potential biofilms in sink drains with disinfectants lacking EPA registration for this use.	
Unresolved Issues		
1.	HCP use of alcohol-impregnated hand wipes is unresolved due to the lack of noninferiority data.^94^	

Note. ABHS, alcohol-based hand sanitizer; CDC, US Centers for Disease Control and Prevention; WHO, World Health Organization; EPA, US Environmental Protection Agency; HCP, healthcare personnel.

### Essential Practices

This section highlights updated recommendations based on evidence that was not available for consideration in the 2014 Compendium. There are 7 essential practices, and 5 of these are previously recommended practices with updated elements. However, 2 practices are new: glove use and prevention of environmental contamination.

The recommendation for promotion of healthy skin and fingernails is reinforced by high-quality evidence and emphasizes the preferential use of alcohol-based hand sanitizer (ABHS) in most clinical situations, which has been shown to be superior to handwashing in preserving healthcare personnel (HCP) hand skin integrity.

1. The document states that policies regarding the use of fingernail polish and gel shellac is at the discretion of the infection prevention program, except among HCP who scrub for surgical procedures, for whom fingernail polish and gel shellac should be prohibited.
2. The document recommends that facilities that are seeking ABHS with ingredients that may enhance efficacy against organisms anticipated to be less susceptible to biocides should consider manufacturers’ product-specific data.
3. The recommendation for placement of ABHS dispensers emphasizes unambiguous and visible accessibility within the workflow of HCP.
4. The document provides minimum thresholds for dispensers to ensure the accessibility of hand hygiene supplies.
5. The document contains additional recommendations for appropriate glove use:
  - HCP should receive competency-based training to ensure knowledge and skill in avoiding contamination during doffing.
  - Routine double-gloving is not recommended, except when specifically recommended for certain job roles or in response to certain high-consequence pathogens.
6. The document contains additional recommendations to reduce environmental contamination associated with handwashing sinks and sink drains.
7. Methods for monitoring adherence to hand hygiene now include direct overt observation, direct covert observation, automated hand hygiene monitoring systems, remote video observation, indirect measures, and audits of accessibility and functionality of supplies. Strengths and weaknesses of each method are discussed in Section 2 and listed in Table 4.

### Additional Approaches

The document maintains the recommendation to wash hands with soap and water during outbreaks of *C. difficile* and norovirus but specifies that ABHS should not be prohibited when caring for patients with *C. difficile* or norovirus. During outbreaks of pathogens of premise plumbing, facilities may consider using a US Environmental Protection Agency (EPA)-registered disinfectant with disinfectant claims against biofilms.

### Unresolved Issues

The recommendation regarding routine use of alcohol-impregnated hand wipes by HCP is unresolved due to the lack of noninferiority data.

## Intended Use

This document was developed following the process outlined in the *Handbook for SHEA-Sponsored Guidelines and Expert Guidance Documents*.^2^ No guideline or expert guidance document can anticipate all clinical situations, and this document is not meant to be a substitute for individual clinical judgement by qualified professionals. This guidance includes methods for the measurement of hand hygiene adherence, maintenance of healthy hand skin and fingernails for HCP, efficacy, and effectiveness of ABHSs, concerns related to outbreaks of waterborne pathogens of premise plumbing, and tools for implementation. This update is applicable to acute-care settings, but the principles and practices described may be indicated in any healthcare setting, including long-term and ambulatory healthcare settings. Hand hygiene is a broad term that includes healthy hand skin and fingernails and methods to clean them: handwashes, scrubs, and rubs. When recommendations are specific to the use of soap and water, the terms “handwash” or “hand scrub” are used. When recommendations are specific to the use of ABHS, the terms “hand sanitizing” or “hand rubbing” are used.

This document is based on a synthesis of evidence, theoretical rationale, current practices, practical considerations, writing-group consensus, and consideration of potential harm, where applicable. A summary of the recommendations is provided in Table 1.

## Methods

The SHEA recruited 3 subject-matter experts in hand hygiene to lead the panel of members representing the Compendium partnering organizations: the SHEA, the Infectious Diseases Society of America (IDSA), the American Hospital Association (AHA), the Association for Professionals in Infection Control, and Epidemiology (APIC), and The Joint Commission, as well as the Centers for Disease Control and Prevention (CDC). The SHEA utilized a consultant medical librarian, who worked with the panel to develop a comprehensive search strategy for PubMed and Embase (January 2012–July 2019; updated in August 2021). Article abstracts were reviewed by panel members in a double-blind fashion through the abstract management software Covidence (Covidence, Melbourne, Australia). The articles were subsequently reviewed as full text. The Compendium Lead Authors group voted to update the literature findings, and the librarian reran the search to update it to August 2021. Panel members reviewed the abstracts of these articles via Covidence and incorporated relevant references.

Recommendations resulting from this literature review process were classified based on the quality of evidence and the balance between desirable and potential undesirable effects of various interventions (Table 2). Panel members met via video conference to discuss literature findings, recommendations, quality of evidence for these recommendations, and classification as essential practices, additional approaches, or unresolved issues. Panel members reviewed and approved the document and its recommendations.

**Table 2.** Quality of Evidence

Quality of Evidence	
High	Highly confident that the true effect lies close to that of the estimated size and direction of the effect. Evidence is rated as “high” quality when a wide range of studies is available with no major limitations, when there is little variation between studies, and when the summary estimate has a narrow confidence interval.
Moderate	The true effect is likely to be close to the estimated size and direction of the effect, but there is a possibility that it is substantially different. Evidence is rated as “moderate” quality when only a few studies are available, when some studies have limitations but not major flaws, when there is some variation between studies, or when the confidence interval of the summary estimate is wide.
Low	The true effect may be substantially different from the estimated size and direction of the effect. Evidence is rated as “low” quality when supporting studies have major flaws, when there is important variation between studies, when the confidence interval of the summary estimate is very wide, or when no rigorous studies are available.

Based on the CDC Healthcare Infection Control Practices Advisory Committee (HICPAC) “Update to the Centers for Disease Control and Prevention and the Healthcare Infection Control Practices Advisory Committee Recommendations Categorization Scheme for Infection Control and Prevention Guideline Recommendations” (October 2019), the Grades of Recommendation, Assessment, Development, and Evaluation (GRADE),^156^ and the Canadian Task Force on Preventive Health Care.^157^

The Compendium Expert Panel, made up of members with broad healthcare epidemiology and infection prevention expertise, reviewed the draft manuscript after consensus had been reached by writing panel members. Following review and approval by the Expert Panel, the 5 partnering organizations, stakeholder organizations, and CDC reviewed the document. Prior to distribution, the guidance document was reviewed and approved by the SHEA Guidelines Committee, the IDSA Standards and Practice Guidelines Committee, and the Boards of SHEA, IDSA, APIC, and The Joint Commission. All members complied with SHEA and IDSA policies on conflict-of-interest disclosure.

## Section 1. Rationale and statements of concern

### Role of hand hygiene in acute care

Hand hygiene has long been a foundational component of infection prevention in all healthcare settings; however, adherence by healthcare personnel (HCP) to hand hygiene protocols has been an ongoing challenge, complicated by the lack of a national standard for measurement and increasingly complex care environments. Furthermore, the proliferation of hand hygiene products in recent decades has created challenges for healthcare administrators and infection prevention leaders to select the most effective, safe, and nonirritating products to support HCP hand hygiene. The purpose of this document is to provide practical guidance, based on up-to-date evidence, for decision making regarding implementation of hand hygiene programs in healthcare facilities.

In the years since publication of the *Strategies to Prevent Healthcare-Associated Infections (HAI) through Hand Hygiene: 2014 Update*, patients receiving healthcare have faced the ongoing threat of infections and antibiotic-resistant organisms potentially spread by contact with the hands of HCP.^3^ Interaction with the healthcare environment can result in hand contamination following activities as brief as touching a bed rail.^4^ Between 2013 and 2019, increases in numbers of patients colonized with extended-spectrum β-lactamase–producing Enterobacterales were noted, while the incidence of carbapenem-resistant Enterobacterales remained stable.^3^ Healthcare facilities experienced the emergence of *Candida auris,* a resistant fungus, and severe acute respiratory syndrome coronavirus 2 (SARS-CoV-2), a novel coronavirus.^5,6^

### State of hand hygiene in acute care

The coronavirus disease 2019 (COVID-19) pandemic was disruptive to infection prevention programs because it precipitated shortages in basic supplies, including ABHS. During the initial phase of the pandemic through December of 2021, the US Food and Drug Administration (FDA) enabled unprecedented use of locally produced ABHS through the provision of temporary guidance allowing for previously unregistered firms to manufacture ABHS.^7^ The pandemic further strained hand hygiene programs because successful implementation relies on HCP input and engagement, which proved challenging throughout a protracted pandemic that resulted in staffing shortages and chronic stress incurred by HCP working in overburdened healthcare systems.

Estimating hand hygiene adherence in the United States is difficult given the variability in facility-specific methods for sampling and measurement. For example, in a point prevalence study conducted in a Canadian intensive care unit (ICU), adherence was reported as 83.5% among nurses and 45.2% among physicians.^8^ In a study in a trauma resuscitation center in the United States, adherence was 7% overall and 0% before a clean procedure.^9^ In the latter study, if direct donning of gloves (ie, without hand hygiene) prior to a clean procedure was considered compliant, the adherence rate would have risen to 57% overall. Clearly there is room for improving adherence and ensuring that hand hygiene programs result in optimal adherence remains a critical element for preventing HAI.

## Section 2. Background on the measurement of hand hygiene adherence

### The goal of measurement

The goal of measuring hand hygiene is to provide timely, meaningful, and actionable feedback to guide HCP improvement. Elements of hand hygiene adherence that are amenable to measurement include the following: adherence to cleaning hands at the right moments before, during, and following care; evaluation of technique; the prevalence of hand dermatitis; and functionality and accessibility of equipment and supplies. Routine measurement should be performed to establish a performance baseline, to support improvement efforts, and to identify barriers and facilitators of adherence. It is unlikely that a single data-collection method will fulfill all the needs of a hand hygiene program.

### Defining opportunities for hand hygiene

Indications for handwashing and hand sanitizing have been clearly defined. The United States Centers for Disease Control and Prevention (CDC) *Core Infection and Control Practices for Safe Healthcare Delivery in All Settings*, published in 2017, is now more closely aligned with the WHO *My 5 Moments for Hand Hygiene* in acute-care settings (Table 3).^10,11^

**Table 3.** Indications for Hand Hygiene

WHO Moments	CDC Indication
1	Immediately before touching a patient
2	Before performing an aseptic task (eg, placing an indwelling device or handling invasive medical devices)
3	After contact with blood, body fluids, or contaminated surfaces
4	After touching a patient
5	After touching the patient environment
	Before moving from work on a soiled body site to a clean body site on the same patient
	Immediately after glove removal
In addition, wash hands when visibly soiled, before eating, and after using the restroom.^a^	

Note. WHO, World Health Organization; CDC, US Centers for Disease Control and Prevention.

a Hand sanitizing with an alcohol-based hand sanitizer is preferred unless handwashing is specifically indicated, or during outbreaks of *C. difficile* or norovirus.

### Methods for measuring adherence

Healthcare facilities may choose from a variety of data collection methods to measure hand hygiene adherence: direct overt observation, direct covert observation, automated adherence hand hygiene monitoring systems (AHHMSs), remote video observation, patient as observer, indirect measurement via product usage, and audits of the functionality and accessibility of equipment and supplies. The measurement method used should be executed in a manner that enhances a culture of safety, results in credible and actionable data, and improves performance toward facility-specific goals. Personnel who conduct hand hygiene observations should be recognized as valued team members and patient safety advocates.^12,13^ Some pitfalls of suboptimal execution of any measurement strategy include biased data, failures to improve adherence, and even the potential for workplace bullying of those collecting observations or reporting results.^13,14^ Strengths and weaknesses of hand hygiene measurement methods are summarized in Table 4.

**Table 4.** Methods to Measure Hand Hygiene

Method	Use	Strength	Weaknesses	Considerations
Direct overt observation^20,153^	Gold standard for evaluation of technique Monitoring prevalence of hand conditions, adherence to facility or unit specific policies Inclusion in prevention bundle checklists can ensure appropriate hand hygiene prior to high-risk procedures (eg, central-line insertion)	Immediate feedback with correction of lapses Those completing prevention bundle checklists are empowered to speak up for patient safety. Can be used as a form of engagement among peers	High risk for bias due to the Hawthorne effect, should not be used to determine rates of adherence during routine care	As part of competency-based training a systematic approach may be used to ensure ongoing, regular assessments of knowledge and skill among all HCP.
Direct covert observation^14,16,17,19,25,47^	Establishment of performance baseline Gauge progress towards facility established goals Evaluation of technique	Barriers and facilitators to hand hygiene can be identified	High risk for observation bias Observations potentially obstructed by physical barriers (eg, curtains) Time and labor intensive Those observed may be skeptical of data Feedback may be delayed or fail to penetrate to those observed Potential for patient harm if lapses not immediately corrected	Facilities should engage in strategies to reduce observer bias. Observers should have clear directions about how to address noncompliance.
Automated hand-hygiene monitoring systems (AHHMSs)	Supplements direct observation Establishment of performance baseline Gauge progress toward facility-established goals Provides trends in hand hygiene performance	More complete data regarding compliance due to continuous monitoring of all shifts and days of week HCP-specific adherence rates can be monitored using some systems Systems may provide real-time reminders to ensure adherence	Unable to evaluate technique Wearable devices may hinder HCP acceptance or completeness of analysis due to noncompliance with wearable use Recording errors may lead to HCP lack of confidence in data, variability in reliability of data between systems and in different physical settings Resource investment is significant and typically recurrent via annual client subscription	Rigorous evaluation is needed to ensure validity. Collaboration with and empowerment of HCP may lead to better acceptance. Will not eliminate need for observation or improvement campaigns but may allow for more targeted interventions
Remote video observation^30,37^	Establishment of performance baseline Gauge progress toward facility-established goals Validate opportunities to determine denominators if not captured by an AHHMS Allows for review of unusual circumstances and validation of other monitoring systems	The absence of a human observer may reduce the Hawthorne effect. Potential for provision of immediate and end-of-shift feedback to individuals and unit managers	Visualization is restricted to camera views	Initial financial burden may be prohibitive. State and local laws and union expectations may complicate implementation. Patient privacy issues must be addressed in policies prior to implementation.
Patient-as-observer^38^	May be appropriate in settings that are challenged with resources for observation such as outpatient settings (eg, emergency department)	Engages and empowers patients to remain aware of and comment on HCP hand hygiene behaviors. May improve patient satisfaction Cost effective	Information is limited to moments included in a single patient contact.	Useful for continuous quality improvement through sharing of patient feedback with HCP
Indirect measures	Event counts Product usage	Allows for assessment of effectively placed dispensers Volume usage may provide trends.	May not correlate with other measurement methods Does not differentiate between roles of HCP versus or healthcare facility visitors	Should not be used as the sole method of measurement
Audits of accessibility and functionality of supplies^43^	Assure infrastructure that supports adherence	Provides assurance of functionality and availability of hand hygiene supplies	Infrastructure may not be amenable to change if restricted by administrative code (eg, building code)	Regular assessment can be performed during routine environment of care rounds.

### Direct overt observation

Direct overt observation (ie, the observer and the observed are known to one another) can be used to evaluate HCP hands for signs of dermatitis, adherence to facility-specific policies for fingernail length, and hand hygiene technique. When included in prevention bundles for high-risk procedures (eg, central-line insertion), direct overt observation can be used to provide immediate feedback, to correct lapses, and to ensure 100% adherence.^15^ Because direct overt observation is inherently subject to bias driven by the Hawthorne effect (ie, deliberate changes in behavior based on the knowledge that one is being observed), it should not be used to determine hand cleaning adherence rates during routine patient care.^16–20^ Targeting direct overt observation to include certain HCP (eg, those performing high-risk procedures like central-line insertion) may be undertaken to ensure that all targeted personnel are observed.

### Direct covert observation

Direct covert observation (ie, the observer is unknown to the observed) is also commonly referred to as a “secret shopper” or anonymous method. Although intended to reduce observer bias, multiple studies have documented observation and selection bias when those under observation became aware of the observer or when certain HCP, shifts, or areas in a unit or facility are oversampled.^21^ In a qualitative study of HCP in 10 acute-care hospitals, observers expressed concern about the Hawthorne effect, and those observed expressed concern about the inability of observers to see their compliance.^14^ In response to a survey about perceptions of hand hygiene monitoring, 58% of 1,120 British HCP did not strongly endorse direct observation for determining hand hygiene adherence.^22^

Methods to reduce bias and improve representativeness of observation include randomly and confidentially scheduling observations during peak activity (eg, morning rounds) and using a systematic method to determine where observations should be collected.^23^ The specific times at which observed opportunities were found to be most representative of all opportunities in a medical ICU were 8 a.m., during morning rounds, 8 p.m., midnight, and 4 a.m. ^24^ To reduce the Hawthorne effect, facilities should consider increasing the frequency of observations, limiting observations to short periods of time (ie, 1–15 minutes), conducting unannounced audits, and enlisting observers unknown to unit personnel.^18,25^

### Automated hand-hygiene monitoring systems (AHHMSs)

The use of AHHMSs has increased since the early 2000s. A variety of systems capture data in several different ways using event counters or wireless connections with dispensers and badges. Data may be collected in an aggregate form (ie, number of activations at room entry) or linked to specific personnel and specific indications. Measurement occurs on all shifts and all days of the week and captures large numbers of hand hygiene opportunities (HHOs), providing insight into adherence patterns.^26,27^ Accuracy may vary, and optimal methods of validating systems have not been identified.^26,28^ Direct observation and remote video observation have been used to estimate denominator data for the calculation of adherence rates that may be used when an AHHMS does not capture HHOs.^29–32^

Real or perceived data inaccuracies generated by AHHMSs may limit the ability to improve hand hygiene. Wearable devices that are unappealing, intrusive, or that interfere with hand hygiene (eg, wristbands) may lead HCP to reject use of the system.^33^ Successful implementation of an AHHMS hinges on leadership commitment and collaboration among the infection prevention team and HCP.^34^ To enhance collaboration, one facility performed structured interviews to obtain HCP feedback about the use and functionality of the system. This intervention resulted in acceptance of the system and sustained improvements in hand hygiene adherence.^35^ The cost of implementation of an AHHMS should be considered, including costs (eg, labor) related to other measurement methods.

### Remote video observation

Remote video observation is a form of direct overt observation in that HCP and patients should be aware that cameras are viewing or recording their hand hygiene behavior. The observer is independent of the unit and unknown to those under observation, which reduces observer and sampling bias.^36^ Remote video observation has been used to validate and benchmark HHOs for use as denominators for certain AHHMSs.^30^ A study coupling remote video observation with rapid and regular feedback to HCP and unit managers resulted in sustained statistically significant improvements in 2 ICUs.^37^ To protect patient privacy the camera view may be restricted to areas of the room in which hand cleaning supplies are located and may have curtains that can be drawn over the lens.^31^ The major challenges of this method include the restricted view of the camera (ie, obstructed at times or limited to areas where hand hygiene is performed), the potential need for patient consent, and financial cost of systems.

### Patient as observer

The patient as observer is another form of direct overt observation that may be useful in outpatient settings when resources for conducting hand hygiene observations are limited. An outpatient clinic using this method asked patients to complete a survey answering a single question, “Did your provider clean their hands before touching you?” More than 75% of patients returned the survey cards and expressed satisfaction with participation in the survey. During an evaluation phase, patient and nurse hand-hygiene auditing data were in concordance 86.7% of the time. HCP received regular individualized and aggregate adherence rates along with patient comments.^38^ This strategy may have limited value in inpatient settings because of variations in patient ability to participate in observations and the increased number of HHOs associated with inpatient care.

### Indirect measurement

Indirect methods of measuring adherence to hand hygiene include volume usage or event counters. Adherence to HHOs based on volume usage is often not attributable to specific department or units within a facility, limiting provision of feedback to HCP. When attempts were made to correlate usage with observational data, researchers noted high compliance by direct observation with no correlation to volume usage. This finding led them to suspect biased observations due to the Hawthorne effect.^39,40^ Using an environmental assessment to identify points of care and an anticipated number of HHOs, indirect methods were used by one facility to determine that the facility would need 200,000 L of hand sanitizer annually to attain high rates of hand hygiene adherence.^41^ This finding may not be helpful for measurement of adherence but may provide insight for emergency planning.

### Audits of the accessibility and functionality of hand hygiene supplies

Regular audits of the accessibility and functionality of hand hygiene equipment and supplies should be conducted to ensure that HCP adherence is supported by the physical environment of care. The National Fire Protection Association requires that ABHS dispensers be tested for proper functioning each time they are refilled.^42^ Audits of equipment can identify broken or empty dispensers or unsuitable sinks, which can then be remediated and tracked so that the facility continuously ensures that HCP have needed supplies to optimize hand hygiene adherence.^22,43^

### Sampling

No national standards have been established regarding the number of observations that should be conducted; however, processes of statistical analysis should be used to determine what constitutes an adequate and representative sample. Methods to estimate the total number of HHOs on various types of inpatient units have included direct observation and video surveillance.^30,44,45^ Facility-wide, an AHHMS recorded between 1.5 and 2.5 million HHOs each month in a 400-bed hospital during the COVID-19 pandemic.^46^ The number of HHOs is highest in ICUs (11.4 per patient hour) and lowest in mother–baby units (3.4 per patient hour).^44^ The mean and median number of HHOs on medical and surgical units were 71.6 and 73.9 HHO per patient day, with a median of 46.7 HHOs on the first shift (7 a.m.–6:59 p.m.) and 28.0 on the second shift (7 p.m.–6:59 a.m.).^30^ When comparing compliance rates obtained by observing the WHO Five Moments to adherence upon entry and exit of patient rooms, a similar adherence rate was observed, indicating that sampling at entry and exit to the room may provide an adequate sample while also decreasing barriers to observation.^24,47^

### Feedback of results

Feedback of measurement results is critical for performance improvement. Goal setting and immediate active feedback have been associated with improved adherence.^48,49^ Feedback is most effective when provided by a supervisor or colleague, when it is provided more than once, when it is given verbally and in writing, and when it is associated with clear targets and action plans.^50^ In a facility that aimed to improve adherence among physicians provided regular reports to chiefs of service, comparative rankings of service varied initially but rose to >90% each month, with sustained improvement over a 2-year period.^51^

Feedback that fails to reach frontline personnel is a barrier to performance improvement.^13,14^ A variety of methods used to provide feedback have included aggregating data and displaying results visually in real time or at the end of a shift. When video feedback of their own performance was confidentially shared with HCP working in hemodialysis, together with written feedback, 7 of the 11 HCP included in the study demonstrated improvement in adherence.^52^ To provide intuitive feedback, a visualization of a handprint with decreasing numbers of bacteria as performance improved, rather than a numeric adherence rate, was presented to HCP. This feedback method did not generate improved performance.^18^ Observation methods are linked to timing of feedback in Table 5.

**Table 5.** Type and Timing of Feedback by Hand Hygiene Measurement Method

Measurement Method	Type of Feedback	Timing of Feedback
Direct overt observations	Individualized	Immediate
Direct covert observations	Individualized	End of observation period
	Aggregate	Regular reports of adherence (eg, weekly)
Automated hand-hygiene monitoring systems (AHHMSs)	Individualized	Immediate (ie, real-time reminders)
	Aggregate	Continuously updated real-time reports
		Regular reports of adherence (eg, weekly)
Remote video observations	Individualized	End of shift
	Aggregate	Regular reports of adherence (eg, weekly)
Patient as observer	Individualized	Regular reports of adherence (eg, weekly)
	Aggregate	Regular reports of adherence (eg, weekly)
Indirect methods	Aggregate	Regular reports of usage or events (eg, monthly, quarterly)

## Section 3. Background on prevention of HAIs through hand hygiene

### Summary of existing guidelines and recommendations

Guidelines for hand hygiene in healthcare settings have been published by the following organizations:

1. Healthcare Infection Prevention Practices Advisory Committee (HICPAC), Centers for Disease Control and Prevention (CDC)^53^
2. The World Health Organization (WHO)^11^
3. The Association for Perioperative Registered Nurses (AORN), related to perioperative hand hygiene^54^
4. The Society for Healthcare Epidemiology of America (SHEA) related to hand hygiene for the operating room anesthesia work area.^55^

### Infrastructure requirements

Hand hygiene programs should have the following elements in place:

1. Accessible and functional hand hygiene supplies, including hand sanitizer dispensers with adequate supplies of ABHS, handwashing sinks, plain or antiseptic handwash, disposable or single-use towels, and hand moisturizer that is compatible with other products and gloves
2. Senior and unit-based leadership support that is responsible and accountable for ensuring engagement and adherence of frontline personnel
3. Infection prevention personnel with training and resources to direct programs aimed at improvement of hand hygiene
4. HCP who have received training to recognize indications for hand hygiene throughout care episodes
5. Trained observers to collect adequate observations to evaluate technique and monitor performance (If automated hand hygiene monitoring systems are used, those charged with their oversight should be skilled in validating data obtained from the system.)
6. Support for data analysis and meaningful communication of monitoring results regardless of the measurement method used by the facility.

## Literature review

### Healthy hand skin and fingernails

Hand hygiene begins with the healthy hands of HCP, defined as being free from pathogenic transient or resident flora, redness, cracks, or wounds, and having short, natural fingernails.^11^ Natural fingernails and those with standard fingernail polish were shown in a single study to be more amenable to cleaning with ABHS than gel or shellac fingernails.^56^ No studies of artificial fingernails or chipped fingernail polish were identified, likely indicating acceptance of previous research related to their association with increased pathogenic flora.^54^ Ongoing exposure to the healthcare environment, water, and antiseptics challenges the barrier integrity of HCP hand skin, placing them at high risk for occupational irritant and allergic contact dermatitis.^57^ In the United Kingdom, 414 (15%) of 2,762 HCP surveyed indicated that their skin had suffered due to work.^58^ Self-reported symptoms of hand eczema were reported by 579 (47%) of 1,232 HCP surveyed in the Netherlands. Among those with hand eczema in the previous 3 months, 84% reported performing their regular duties at least 1 day while symptomatic.^59^

Allergens associated with hand hygiene include antiseptics, latex, rubber accelerators, fragrances, surfactants, and preservatives.^60^ Increasing exposure of HCP to chlorhexidine gluconate (CHG), in both HCP hand hygiene products, and in patient bathing products has stimulated research examining the potential for sensitization of HCP to CHG. In a cross-sectional survey of >1,000 nurses, >70% reported using chlorhexidine >20 times per shift.^61^ Also, 114 (30.7%) of these nurses experienced symptoms of sensitization and regularly experienced self-reported symptoms included dry skin (86.7%), localized rash (73.3%), and wheezing or coughing (20.6%). No anaphylactic events were reported. Hand hygiene programs should implement strategies to engage HCP in primary and secondary prevention of hand eczema, and allergic or irritant dermatitis.^62–64^

## Hand hygiene product safety and efficacy

### Regulatory background

In December 2017, the FDA’s tentative final monograph for over-the-counter healthcare antiseptic drug products, initially published in 1994, and amended in 2015, was finalized.^65^ This rule established active ingredients for over-the-counter use as HCP handwashes or surgical hand scrubs, HCP hand rubs and surgical hand rubs. Pending additional data to establish generally recognized as safe and effective (GRAS/GRAE) determinations, the FDA deferred regulatory action on 6 antiseptics included in the final monograph: benzalkonium chloride, chloroxylenol, ethyl alcohol, isopropyl alcohol, and povidone iodine.^65,66^ The FDA requires manufacturers to meet current safety standards related to human safety, nonclinical safety (eg, reproductive, toxicity studies), potential hormonal effects, and antimicrobial resistance. Although still eligible for inclusion in HCP handwash, triclosan was removed from the consumer antiseptic monograph due to potential hormonal effects and potential contribution to antimicrobial resistance.^67^ CHG was introduced in the United States after the 1994 tentative final monograph was published and was determined to be ineligible for inclusion; products formulated with CHG continue to be regulated as new drugs.

### Safety

In response to the FDA request for up-to-date safety data, observational studies aimed at estimating maximal use of ABHS by HCP have been conducted. Studies using electronic event counting have reported wide variation in usage frequency depending on role and service (eg, nurse vs physician and operating room vs medical ICU). Nurses likely have the most frequent exposure to hand-sanitizing formulations, and the maximal use among 95% of nurses working 12-hour shifts has been reported as 15 uses per hour or 141 uses per shift.^68^

### Potential for absorption of ABHS

Dermal absorption of alcohol has been considered a potential reproductive risk for women as there are no established minimum safe levels of alcohol exposure during pregnancy. Researchers conducting a safety assessment noted that ABHS can result in very low but detectable internal doses that approximate those associated with consumption of nonalcoholic beverages (eg, juices that may undergo natural fermentation). The authors concluded that the benefits of preventing infection by using ABHS outweigh risks of maternal exposure to alcohol through dermal absorption.^69^

Respiratory absorption of alcohol was examined among preterm neonates in isolettes. Volatilized ethanol from a single exposure resulted in a detectable level of blood alcohol (0.036 mg/dL), lower than the European Medicines Agency limits for ethanol exposure in children.^70^ Exposures of neonates could be reduced by ensuring drying of ABHS prior to placing hands within an isolette. Evidence about developmental toxicity related to antiseptics other than alcohol is limited. Respiratory absorption of alcohol was also studied among anesthesiologists; 8 of 130 breathalyzer tests were positive within 2 minutes of use of ABHS, and none resulted in a positive blood alcohol reading.^71^

### Adverse events

The literature review did not yield any reports of serious harm associated with the use of ABHS in healthcare settings. Two cautionary reports from community settings may have relevance for healthcare facilities. The first involved failures in the manufacture of an ABHS produced outside the United States and distributed during the SARS-CoV-2 pandemic. An FDA consumer alert warning about hand sanitizers that contained methanol resulted in 2 states reporting 15 cases of methanol poisoning following intentional consumption. Methanol poisoning resulted in 4 deaths and permanent disabilities in survivors.^72^ In areas in which intentional consumption is an identified risk (eg, behavioral health units) measures to maintain control of ABHS should be taken.

The second harmful event occurred outside the United States and involved unsafe dispensing of ABHS. The height of the dispensers and the method of activation (eg, a foot pump) allowed ABHS to be directed toward the faces of small children, resulting in splashing of the eyes and severe ocular injuries.^73,74^ In the United States, the National Fire Protection Association requires dispensers be designed so that accidental activation is minimized and requires that dispensers be tested each time a new refill is installed. This may be particularly important in pediatric facilities.^42^ All local administrative codes related to fire safety when choosing locations for installation of ABHS dispensers and storage of refills should be followed. Pediatric facilities should evaluate the height of wall-mounted ABHS dispensers and the placement of pump bottles to avoid activation by young children, and adult supervision should be ensured.

### Efficacy of hand hygiene formulations

The current approval process for antiseptic drug products does not allow manufacturers to make organism or disease-specific prevention claims, and results of efficacy studies may be difficult to compare. Premarket assurance of efficacy is determined using methods published by the American Society for Testing and Material (ASTM) or internationally using the European Norm.^75^ These methods generally evaluate bacterial log reduction on artificially contaminated hands or finger pads. Handwashes are tested using a 5-mL dose, and hand rubs are tested using a 1.5-mL dose or a single impregnated wipe. Test organisms are either gram-negative organisms (eg, *Serratia marcescens* and *Escherichia coli*) or gram-positive organisms (eg, *Staphylococcus aureus*). The ASTM recommends methods of efficacy testing against viruses and fungi, but these are not required prior to distribution in the United States.^11^

## Efficacy of ABHS

### Viral pathogens

Prior to the pandemic, suspension testing evaluating the efficacy of WHO hand-rub formulations found that both formulations inactivated Ebola and emerging coronaviruses with a 30-second exposure time.^76^ A study investigating efficacy against adenovirus serotypes 8, 19, and 37, typically associated with epidemic keratoconjunctivitis, reported a 2.5 log_10_ reduction with combinations of alcohol and lower reductions when alcohol was combined with CHG.^77^

A systematic review of 56 studies testing efficacy of ethanol against viruses found high efficacy against enveloped viruses and less efficacy against nonenveloped viruses. Efficacy against nonenveloped viruses was improved when acids were added to alcohol-based formulations.^78^ This finding is consistent with other studies showing that excipient ingredients (ie, those other than the active ingredient) can enhance or reduce efficacy of alcohol such that in certain formulations, lower volumes of ethanol may produce higher reductions in bacteria than formulations with higher ethanol concentrations.^79^

### Candida auris

A study examining germicidal activity of hand-sanitizing preparations against *C. auris* demonstrated that a 70% ethanol-based hand sanitizer resulted in a 4 log_10_ reduction in organism when tested using a quantitative carrier method.^80^ Surgical hand scrubs containing CHG resulted in <2.0 log_10_ reduction and were less efficacious when alcohol was not included in the formulation. Researchers also investigated 2 alcohol-based formulations: a combination of ethanol (54%–66%)–isopropyl (9%–11%) and a 75% ethanol sanitizer against *C. auris* on artificially contaminated pig skin. The formulations reduced organism load by 2.92 log_10_ and 2.44 log_10_, respectively.^80^

### *Vancomycin-resistant* Enterococcus *(VRE)*

Isolates from a single healthcare institution exhibited tolerance when exposed to 23% (v/v) isopropanol.^81^ When these isolates were exposed to isopropyl alcohol at 70% (v/v) in a broth culture, complete killing and an 8 log_10_ reduction was obtained. These researchers hypothesized that as tolerance increases exposure of *E. faecium* to less than the maximum biocide concentration could select for increasing tolerance. These findings emphasize the importance of adequate formulations and appropriate real-world applications.

### Efficacy of benzalkonium chloride

The persistent activity of benzalkonium chloride (BK), a quaternary-ammonium compound, either alone or in combination with alcohol, has been described. When *S. aureus* was used as test organism, BK alone produced log_10_ reductions up to 4 hours after application.^82,83^ BK 0.2% produced >3 log_10_ reductions in SARS-CoV-2 within 15 seconds of exposure.^84^ Evidence against gram-negative organisms is lacking and concerns remain about the intrinsic resistance of *Burkholderia cepacia* complex to BK.^85^ Due to the organism’s ability to adapt to nutrient-depleted solutions, *B. cepacia* complex poses a risk for contamination of non–alcohol-based hand sanitizers.^86^ In 2020, a recall of non–alcohol-based hand sanitizer followed product contamination with *B. cepacia* complex from the municipal water supply used in production.^87^ Non–alcohol-based hand sanitizers should not be used in clinical settings.

### Effectiveness in clinical use

Real-world hand-hygiene effectiveness is related to product formulation, application volume, thorough application to all hand surfaces, and rates of personnel adherence.^88–90^ In a network modeling study describing methicillin-resistant *S. aureus* (MRSA) colonization rates among neonatal ICU (NICU) patients, colonization was reduced as hand hygiene adherence increased.^88^ Even under optimal conditions, the most vulnerable patients may still acquire pathogens; therefore, HCP should aim for high adherence to each element of hand hygiene.

A randomized control trial examined the effectiveness of 3 hand hygiene protocols comparing ABHS application to all hand surfaces, ABHS application using a WHO-recommended structured hand rub and chlorhexidine gluconate (CHG) handwash. ABHS was as effective as the CHG handwash in reducing bacteria on the hands, and ABHS application was the most time-efficient means of performing hand hygiene.^91^ This study was replicated using MRSA as a test agent, and ABHS was as effective as CHG handwash in real-world use.^92^ Residual effectiveness has been demonstrated with formulations that combine alcohol with either 2%–5% CHG or 0.1% BK.^82^ Such formulations may be beneficial particularly if used in high-risk areas (eg, ICU or transplant units) or prior to invasive procedures like central-venous access.

### Mode of delivery

ABHS is available in several delivery forms such as liquid, gel, foams, and wipes. Alcohol-impregnated wipes were previously reported to have similar efficacy to gel and foam hand rubs when influenza virus was the organism of interest.^93^ In a study using *E. coli* as the test organism to compare ABHS hand rubs to cotton or polypropylene hand wipes, hand rubs were superior to hand wipes.^94^ Further testing is needed to determine noninferiority of alcohol-impregnated hand wipes to hand rubbing with ABHS.

### Effective volume and dose

The volume of hand sanitizer or antimicrobial handwash formulations may be considered a dose, and the dose must be sufficient to cover all surfaces of the hands. Touch-free dispensers provide a mean dose ranging from 0.6 mL to 1.3 mL with a mean drying time of 12–22 seconds.^89^ For persons with large hands, a dose of 4–6 mL may be needed to achieve >2 log_10_ reductions in bacteria on the hands.^95^ To obtain antisepsis, the volume of ABHS should be customized according to the size of the individual’s hands. This volume may be communicated as a “palmful” of hand sanitizer and may require more than a single activation. Some AHMMSs measure only 1 activation if multiple dispenser activations occur during brief time spans (eg, 2 seconds), which may be important in avoiding the overestimation of compliance if event counters are used for measurement.

### Effective technique

Techniques for both handwashing and hand sanitizing should focus on coverage of all hand surfaces for an appropriate length of time. This literature review did not identify studies examining the duration of handwashing; a minimum of 15 seconds of scrubbing for routine handwashing has been previously recommended by the CDC.^53^ Studies have reported no difference in bacterial load when comparing hand-rub durations of 15 and 30 seconds, but adherence increased by 27% with shorter hand rubs.^96,97^ When technique was included with observations of adherence to indications for hand hygiene, only 7% of HCP attained full coverage of all hand surfaces; the thumb and fingertips were the most frequently missed areas of the hands.^98^ Attainment of full hand-surface coverage while rubbing for 15 seconds or longer should be included in HCP evaluations of hand-hygiene technique.^96,97^

### Organisms with less susceptibility to biocides

Spore-forming organisms (eg, *C. difficile* and *B. cereus*) and small nonenveloped viruses (eg, norovirus) are difficult to inactivate with surface disinfectants and may not be inactivated by alcohol. Many facilities lack clarity regarding whether alcohol-based hand sanitizer should be used when contact with organisms that are less susceptible to biocides occurs. These organisms may also be difficult to remove through handwashing.^99^ Using a nontoxigenic strain of *C. difficile* to test reductions associated with handwashing technique a 1.3 log_10_ reduction was attained with an unstructured handwash and a 1.7 log _10_ reduction was attained using a structured handwash (ie, the WHO How to Handrub).^100^

When exposure to potential spore-forming organisms or small nonenveloped viruses is anticipated (ie, patients diagnosed with *C. difficile* infection or norovirus or those with new acute diarrhea or vomiting), the CDC recommends standard and contact precautions for all contacts with the patient and their surroundings. HCP need clear messaging about hand hygiene in response to these organisms. Those directing hand hygiene programs should do the following: (1) help HCP remain aware of organisms with biocide resistance that are circulating in the facility; (2) emphasize the importance of reducing hand contamination through the use of gloves according to standard and contact precautions; (3) maintain the availability of ABHS in the presence of these organisms; (4) in all settings, regardless of organisms present, always wash hands if visibly soiled, before eating, and after using the bathroom; and (5) emphasize the importance of thorough hand cleaning with the consideration of educating HCP in WHO-structured techniques for handwashing and hand sanitizing.^101^

### Accessible hand hygiene supplies

Among 350 HCP surveyed in the United States and Canada, lack of access to supplies was described as the primary barrier to adherence.^101^ The physical infrastructure required to implement hand hygiene in all facilities consists of access to ABHS and handwashing stations supplied with water, soap (ie, plain or with an antiseptic), towels, gloves, and hand moisturizers that are compatible with antiseptics and gloves. In perioperative and procedural areas, including ICUs, surgical hand scrub and surgical hand rub should also be accessible to HCP.

### Supplies for hand sanitizing

Several studies have examined optimal placement of ABHS dispensers within HCP workflow. On a general inpatient unit, sequentially increasing the number of wall-mounted ABHS dispensers above a minimum threshold (defined as 2 dispensers per room, 1 dispenser in the hallway, and 1 dispenser in the patient room) did not result in improved adherence.^102^ More than half of hand hygiene events occurred in the hallway. Once inside the room 75% of events involved dispensers just inside the doorway. In multipatient rooms (eg, bays), a threshold for accessibility was considered 1 dispenser for every 2 beds.^48^ Ensuring accessibility to ABHS was most difficult when workflows involved crowded spaces with no dedicated bed space (eg, hallway care). In these spaces, a focus on the WHO Five Moments and the CDC indications prior to and immediately following patient care may be helpful. When patients cannot be housed in rooms, facilities should ensure that the patient zone is clearly defined and that hand hygiene supplies are within reach.^103,104^

In addition, ABHS dispensers that are clearly identifiable (ie, distinct from soap or moisturizer dispensers), function as visual cues to perform hand hygiene. Improved adherence to hand hygiene when ABHS dispensers are visible and accessible within the workflow of HCP has been replicated in multiple healthcare and specialty settings and among inpatient and outpatient areas.^105–111^ Event counters can be used to establish the best location for dispensers on individual units and within patient rooms.^112^ When units are well equipped with mounted dispensers, individual pocket-sized dispensers did not increase adherence to hand hygiene, possibly because individual-sized dispensers are more difficult to use than wall-mounted dispensers.^113^ When ABHS dispensers cannot be wall mounted, and there is no risk of intentional ingestion, pump bottles can be mounted on beds or placed on bedside tables, work surfaces, and other locations in the workflow of personnel.

### Supplies for handwashing

Accessibility and visibility of sinks affects HCP adherence to handwashing. Sinks visible from the point of care, rather than sinks that are separated from the point-of-care by a wall or door, resulted in more frequent handwashing with longer duration, particularly if visible from occupied beds.^114^ Following care of individuals with *C. difficile* infection, proper timing of glove removal upon leaving the patient zone was directly associated with hand washing, whereas increasing distance of the sink was inversely associated with handwashing compliance.^115^

### Contamination of water and plumbing

Contamination of supply or wastewater (ie, biofilms within sink drains) with waterborne pathogens of premise plumbing may increase opportunities for the environmental contamination of HCP hands, clothing, and patient care supplies.^114,116–123^ Splashing of water and aerosolization of organisms has included extended-spectrum β-lactamase–resistant organisms, Enterobacterales, *Elizabethkingia meningoseptica,* KPC-2–producing *Klebsiella* spp, and *Pseudomonas* spp.^116,120^ In an observational study analyzing behavior at sinks in an ICU, handwashing occurred in only 4% of the total interactions with the sink. Other activities included filling and emptying of water glasses, medication cups, and tube feed bags, draining IV bags, preparation and discarding of food and beverages and placement of patient care items on nearby countertops.^124^ Slow drainage from sinks was also noted to result in increased contamination of the sink bowl and nearby surfaces.^118^ Contamination was reduced when sink use was restricted to handwashing and basins were disinfected daily with bleach.^123^ In 2017, the United States Environmental Protection Agency released regulatory guidance for pesticidal claims against biofilm bacteria on hard, nonporous surfaces including sink drains.^125^ As of 2022, several pesticides have been registered, although there are no protocols for use or optimal intervals for drain cleaning have been published. Handwashing sinks should be included in water infection control risk assessments. Resources are available on the CDC Reduce Risk from Water webpage.^126^

### Contamination of supplies

Contamination of soap and ABHS dispensers is less frequently described. However, ABHS dispensers have been found to be contaminated with *Staphylococcal* spp, this contamination increases with increasing use of the dispensers.^114^ During the SARS- CoV-2 pandemic, the CDC advised against refilling (or “topping off”) dispensers intended for single use, acknowledging a lack of studies but the potential for introducing spore-forming organisms.^127^ Higher rates of mechanical defects were reported among touchless ABHS dispensers compared to mechanical dispensers, suggesting the potential need for ongoing maintenance.^128^ Using a crossover design, researchers examined the use of jet air dryers compared with single-use towels. The target organisms (methicillin-susceptible *S. aureus,* methicillin-resistant *S. aureus*, enterococci, and extended-spectrum ß-lactamase–producing bacteria) were recovered from washroom floors and from the surfaces of jet air dryers. These researchers concluded that single-use towels had less propensity for environmental dispersion of organisms and that jet air dryers were not acceptable for clinical use.^129^

### Nonsterile glove use

Use of nonsterile gloves is inextricably linked to hand hygiene, not only providing benefits like reduced hand contamination during care but also introducing risks such as increased hand contamination during doffing and increased contamination of the patient care environment. Studies evaluating the transfer of environmental contaminants to gloves and bare hands have reported a reduction in hand contamination when gloves are worn. The microbial load of gloved and bare hands stabilized after 4–6 contacts within a patient environment; gloved hands having a microbial load 4.7% lower than bare hands.^130^ Hand contamination increased when gloves fit poorly (ie, were too large), likely due to increased exposed surface area. Transfer efficiencies of *A. baumannii* when latex gloves were worn reduced fomite-to-fingerpad transfer by 56% and reduced fingerpad-to-fomite transfer by 47%.^131^ As anticipated, failure to wear gloves was independently associated with hand contamination following care of patients with *C. difficile* infection.^132^

In a randomized clinical trial studying the impact of nonsterile glove use after hand hygiene versus hand hygiene alone for all care, no significant difference was detected in late-onset invasive infection or necrotizing enterocolitis in neonates cared for by HCP in these 2 groups. Significantly fewer gram-positive bloodstream infections and central-line–associated bloodstream infections occurred among neonates whose care providers donned gloves after hand hygiene.^133^ This finding suggests that hand hygiene plus donning nonsterile gloves prior to patient and vascular device contact may lessen the risk of infection among units that care for preterm neonates.

Numerous barriers to hand hygiene prior to use of nonsterile gloves have been reported. Frequently observed noncompliant behavior includes reductions in hand hygiene prior to patient contact and failures to change gloves at appropriate moments.^134^ In a qualitative study, HCP reported that donning gloves on wet hands was unpleasant and that donning gloves without hand hygiene immediately prior saved time, particularly if anticipated contact was brief (eg, delivering a patient food tray). Physical barriers, such as lack of ABHS access at points where gloves are donned, also resulted in nonadherence.^135^ In an ICU, when hand hygiene prior to donning gloves was compared to direct gloving (ie, no hand hygiene prior to donning nonsterile gloves), there was no significant difference in the average CFU on the surface of the gloves (6.9 vs 8.1 CFU, respectively).^136^ It took an average of 31.5 extra seconds to perform hand hygiene before donning gloves, which equates to ∼20 minutes of extra time for the average ICU nurse caring for a patient in contact isolation during a 12-hour shift. Neither the CDC nor the WHO consider donning nonsterile gloves to be an indication for hand hygiene, but it is frequently associated with Moment 1 of the WHO My 5 Moments and the CDC indication for hand hygiene prior to patient contact. Infection preventionists and hospital epidemiologists should evaluate the potential impact to patient and HCP safety associated with direct gloving to determine whether it may be considered compliant according to facility policies.

Inappropriate glove use, during tasks when there is no risk of exposure to infectious matter, or failures to change gloves at appropriate moments during care, has been associated with environmental contamination.^135,137^ In an observational study, the patient care items most frequently touched by soiled gloves included disinfectant wipes or packaging of patient-care items, patient skin, patient clothing, and durable medical equipment.^138^

In a study of outpatient wound-care providers, hand contamination with a pathogen following doffing of gloves was documented in 10 (19.6%) of 51 encounters.^139^ Simulations of doffing using fluorescent gel indicated that the fingertips and wrists were the areas of the hands most likely to be contaminated. Hand contamination was reduced when doffing was modified to include removal of the first glove without touching the hand, followed by inserting the fingers into the dorsal side of the remaining gloved hand to slide the glove off the hand.^140^

Double gloving has been proposed to further reduce hand contamination. In a study examining double gloving using a nonenveloped viral surrogate for Ebola, the inner gloves of 8 of 15 participants were contaminated. One participant who did not have inner glove contamination had hand contamination. These researchers concluded that random contamination events can occur even when double gloving is used, and they emphasized the importance of hand hygiene after doffing.^141^

Incorporation of disinfection of gloves with ABHS during task saturated clinical care has also been investigated to reduce hand contamination associated with glove use. These studies were performed independent of glove manufacturers. In one study, disinfection of gloves during care episodes led to increased adherence to hand hygiene.^142^ Exposure to ABHS did not impact tensile strength of nitrile gloves; however, risk of perforation increased when gloves were worn continuously for >15 minutes following wound dressing changes and following patient or resident bathing activities.^143^ Researchers also examined disinfection of gloves using bleach wipes prior to doffing; this reduced hand contamination significantly but generated concerns about respiratory irritation associated with use of bleach wipes.^144^ Disinfection of gloves prior to doffing is included in CDC guidance on PPE use in response to certain high-consequence pathogens.^145^

Wearing gloves for long periods during a work shift increases the risk of occupational irritant or allergic dermatitis. When evaluating allergic dermatitis, it is important to consider ingredients used in the manufacture of gloves, such as rubber accelerators that are used in the manufacture of nitrile gloves.^60^ Given the risk and benefits associated with glove use, a balanced approach is needed. HCP should be instructed in appropriate use of gloves, facility expectations related to hand hygiene prior to donning gloves, when to change gloves during care, and methods of doffing to reduce hand contamination. Ongoing observations of glove use, donning and doffing as indicated, with immediate performance of hand hygiene following doffing, should be conducted when monitoring adherence to hand hygiene. Fluorescent gel applied to gloves prior to doffing can be a useful tool to educate personnel about hand contamination during doffing.^140^

### Presurgical hand antisepsis

The purpose of a surgical hand scrub or rub is to reduce transient and resident organisms on the hands for the duration of the operative procedure. The persistent activity of the surgical hand rubs or scrubs is a key feature of these antiseptics.^66^ Waterless surgical hand rubs provide bacterial reductions that are no different than those provided by surgical hand scrubs and are less damaging to the skin.^146–148^ Previous research has shown that scrubbing with a brush may damage skin and increase bacterial shedding from the hands.^11,149^

In a quasi-experimental study using direct overt observation to ensure full compliance with the WHO surgical hand scrub technique, alcohol-based hand scrub improved quality and reduced the duration of the preparation with no significant change in surgical-site infection rates.^150^ When comparing a surgical hand scrub formulated with chlorhexidine, waterless surgical hand rub, and povidone iodine, both the CHG and waterless surgical hand scrub had greater reductions of colony-forming units on the hands than povidone iodine. These researchers concluded that preference, compliance, and cost are key to selection of products for presurgical hand antisepsis.^147^

Scrubbed personnel should pay attention to the amount of waterless product dispensed and increase amounts if needed. Manufacturers often recommend 4–6 mL alcohol-based surgical hand scrub, but individuals with larger hands and forearms may need to use higher volumes.^151^ The volume used should keep the skin wet for the duration of the surgical hand rub recommended by the manufacturer.

The CDC has recommended against the wear of artificial fingernails or extenders in high-risk areas and makes no statements about jewelry. Cochrane reviewers were able to identify only 1 study comparing wear of freshly applied fingernail polish, old or chipped fingernail polish, and natural fingernails and no random control trials evaluated jewelry. This finding may indicate that prohibitions against wearing of fingernail polish or jewelry by personnel scrubbed for surgical procedures is an accepted practice, and studies involving randomization may pose ethical concerns.^152^ Because scrubbed personnel are actively interacting with the sterile field, we recommend that fingernails be maintained without polish.

Educational interventions may improve compliance with surgical hand scrubs and surgical hand-rub performance.^150,153^ Structured methods for scrubbing may result in improved technique. Direct overt observation can be used to evaluate technique and to correct lapses during surgical scrubbing. This intervention includes observing for sufficient coverage of arms and adequate time spent performing the scrub.^153^ Overt or covert observation may be used to assess ergonomic adjustments needed such as ensuring access to products or placing timers in view of the scrub sink. Fluorescent indicators have been valuable for instructing personnel in proper scrub technique.^150^

## Section 4. Recommended strategies to improve hand hygiene

Recommendations are categorized as either (1) essential practices that should be adopted by all acute-care hospitals or (2) additional approaches that can be considered in locations and/or populations within hospitals when they are experiencing an outbreak or when HAIs are not controlled despite full implementation of essential practices. Essential practices include recommendations in which the potential to prevent HAIs outweighs the potential for undesirable effects. Additional approaches include recommendations in which the intervention is likely to reduce risk of HAIs, but concern remains regarding the risks for undesirable outcomes, recommendations for which the quality of evidence is low, recommendations in which cost-to-benefit ratio may be high, or recommendations in which evidence supports the impact of the intervention in select settings (eg, during outbreaks) or for select patient populations. Hospitals can prioritize their efforts by initially focusing on implementation of the prevention strategies listed as essential practices. If surveillance or other risk assessments suggest ongoing opportunities for improvement, hospitals should consider adopting some or all of the prevention approaches listed as additional approaches. These can be implemented in specific locations or patient populations or can be implemented hospital-wide, depending on outcome data, risk assessment, and/or local requirements. Each infection prevention recommendation is accompanied by a quality-of-evidence grade (Table 2).

### Essential practices for preventing HAIs through hand hygiene

1. **Promote the maintenance of healthy hand skin and fingernails.** ^10,57,58,154^ (Quality of evidence: HIGH)
  - Promote the preferential use of ABHS in most clinical situations.^10,64^ (Quality of evidence: HIGH)
  - Perform hand hygiene as indicated by the CDC or the WHO Five Moments (Table 3). (Quality of evidence: HIGH)
  - Include fingernail care in facility-specific polices related to hand hygiene:
    - HCP should maintain short, natural fingernails.
    - Fingernails should not extend past the fingertip.
    - HCP who provide direct or indirect care in high-risk areas (eg, ICU, perioperative) should not wear artificial fingernail extenders.
    - Prohibitions against fingernail polish (standard or gel shellac) are at the discretion of the infection prevention program, except among scrubbed individuals who interact with the sterile field during surgical procedures; these individuals should not wear fingernail polish or gel shellac.
  - Include measures for primary and secondary prevention of dermatitis.
  - Provide HCP with readily accessible, facility-approved hand moisturizers.^64^
  - Engage all HCP in primary prevention of occupational irritant and allergic contact dermatitis.^62–64,154,155^ (Quality of evidence: HIGH)
    - Primary prevention of HCP dermatitis should include HCP education about the following:
      - Strategies to maintain healthy hand skin
      - Handwashing techniques to promote healthy hand skin, such as avoiding hot water and patting rather than rubbing hands dry
      - When and how to use gloves, change gloves, take periodic breaks to allow hands to dry, and routinely apply facility-approved moisturizers^62^
      - The potential for allergic reactions to components in ABHS formulations, antiseptics (eg, CHG), glove material, or products used during these products’ manufacture (eg, accelerants)^60,158^
    - Provide facility-approved hand moisturizer that is compatible with antiseptics and gloves^64^
    - Evaluate new products for the absence of potential allergenic surfactants, preservatives, fragrances, or dyes^60^
    - Workplace self-screening for dermatitis^159,160^
    - Refer HCP to the occupational health department for assistance in cases of hand eczema or dermatitis
  - Provide cotton glove liners for HCP with hand irritation and educate these HCP on their use (ie, following instructions for use, laundering, and/or discarding).^64^ (Quality of evidence: MODERATE)
2. **Select appropriate products.** (Quality of evidence: HIGH)
  - For routine hand hygiene, choose liquid, gel, or foam ABHS with at least 60 % alcohol.^8,10,65,76,79,94^ (Quality of evidence: HIGH)
  - Involve HCP in the selection of products.^147^ (Quality of evidence: HIGH)
  - Obtain and consider manufacturers’ product-specific data if seeking ABHS with ingredients that may enhance efficacy against organisms anticipated to be less susceptible to biocides.^78,79^ (Quality of evidence: MODERATE)
  - Confirm that the volume of ABHS dispensed is consistent with the volume shown to be efficacious.^89,95,98^ (Quality of evidence: HIGH)
  - Educate HCP about the appropriate volume of ABHS and the time required to be effective.^95^ (Quality of evidence: HIGH)
    - The volume of hand sanitizer should be sufficient to cover all surfaces of the hands and may require >1 dispenser actuation for large hands.^95^ (Quality of evidence: HIGH)
    - When sanitizing, HCP should rub hands for a minimum of 15 seconds. When handwashing, HCP should scrub for a minimum of 15 seconds.^53,96,161,162^ (Quality of evidence: HIGH)
    - Facilities should consider fluorescent indicators for use when training HCP in the application of ABHS and handwashing.
  - Provide facility-approved hand moisturizer that is compatible with antiseptics and gloves.^64^ (Quality of evidence: HIGH)
  - For surgical antisepsis, use an FDA-approved surgical hand scrub or waterless surgical hand rub. (Quality of evidence: HIGH)
    - Complete surgical hand antisepsis by performing a surgical hand rub or surgical hand scrub. (Quality of evidence: HIGH)
    - Scrub brushes should be avoided because they damage skin. (Quality of evidence: HIGH)
3. **Ensure the accessibility of hand hygiene supplies.** (Quality of evidence: HIGH)
  - Ensure that ABHS dispensers are unambiguous, visible, and accessible within the workflow of HCP.^105–111^ (Quality of evidence: HIGH)
    - Use a systematic method (eg, workflow evaluation, event counters) to determine optimal placement of ABHS dispensers. (Quality of evidence: HIGH)
  - In private rooms, consider 2 ABHS dispensers per private room the minimum threshold for adequate numbers of dispensers: 1 dispenser in the hallway, and 1 dispenser in the patient room.^102^ (Quality of evidence: HIGH)
  - In semiprivate rooms, suites, bays, and other multipatient bed configurations, consider 1 dispenser per 2 beds as the minimum threshold for adequate numbers of dispensers. Place ABHS dispensers in the workflow of HCP.^48^ (Quality of evidence: LOW)
  - Ensure that the placement of hand hygiene supplies (eg, individual pocket-sized dispensers, bed mounted ABHS dispenser, single use pump bottles) is easily accessible for HCP in all areas where patients receive care.^103,104^ (Quality of evidence: HIGH)
  - Evaluate for the risk of intentional consumption. Utilize dispensers that mitigate this risk, such as wall-mounted dispensers that allow limited numbers of activations within short periods (eg, 5 seconds). (Quality of evidence: LOW)
    - If individual pocket-sized dispensers are used when caring for individuals at risk for intentional consumption, they must always remain in the control of the HCP.
  - Have surgical hand rub and scrub available in perioperative areas. (Quality of evidence: HIGH)
  - Consider providing ABHS hand rubs or handwash with FDA-approved antiseptics for use in procedural areas and prior to high-risk bedside procedures (eg, central-line insertion). (Quality of evidence: LOW)
4. **Ensure appropriate glove use to reduce hand and environmental contamination.** ^130–132,138^ (Quality of evidence: HIGH)
  - Use gloves for all contact with the patient and environment as indicated by standard and contact precautions during care of individuals with organisms confirmed to be less susceptible to biocides (eg, *C. difficile* or norovirus).^10^
    - HCP caring for preterm neonate with central lines should perform hand hygiene before donning nonsterile gloves prior to patient and vascular device contact.^133^ (Quality of evidence: HIGH)
  - Educate HCP about the potential for self-contamination and environmental contamination when gloves are worn. (Quality of evidence: HIGH)
    - Whenever hand hygiene is indicated during episodes of care, HCP should doff gloves and perform hand hygiene.
  - Clean hands immediately following glove removal. If handwashing is indicated and sinks are not immediately available, use ABHS and then wash hands as soon as possible.
  - Educate and confirm the ability of HCP to doff gloves in a manner that avoids contamination. (Quality of evidence: HIGH)
    - Consider using fluorescent indicators applied to gloves during demonstrations of doffing to help HCP visualize how contamination may occur.
5. **Take steps to reduce environmental contamination associated with sinks and sink drains.** ^114,116–123^ (Quality of Evidence: HIGH)
  - Ensure that handwashing sinks are constructed according to local administrative codes.
  - Include handwashing sinks in water infection control risk assessments for healthcare settings.
  - If possible, dedicate sinks to handwashing.
  - Educate HCP to refrain from disposing substances that promote growth of biofilms (eg, intravenous solutions, medications, liquid food, or human waste) in handwashing sinks.
  - Use an EPA-registered hospital disinfectant to clean sink bowls and faucets daily.
  - Do not keep medications or patient care supplies on countertops or mobile surfaces that are within 1m (3 feet) of sinks.
    - Install splash guards if countertops must be used to store supplies.
  - Provide disposable or single-use towels to dry hands. Do not use hot air dryers in patient care areas.
  - Consult with state or local public health officials when investigating confirmed or suspected outbreaks of healthcare-associated infections due to waterborne pathogens of premise plumbing.
6. **Monitor adherence to hand hygiene.** (Quality of evidence: HIGH)
  - Use multiple methods to measure adherence to hand hygiene.
  - Consider advantages and limitations of each type of monitoring.
    - Direct observation
      - Direct overt observation^20,153^
        1. To evaluate and improve HCP technique and adherence to facility-specific policies
        2. To prevent lapses during high-risk procedures such as insertion of invasive devices.
      - Direct covert observation^14,16,17,19,25,47^
        1. To monitor rates of adherence
        2. To elucidate contextual barriers and facilitators to hand hygiene
        3. To provide corrective feedback to individuals.
  - Use a systematic approach to determine where and when observations should occur.^23,24^
    - Provide training for individuals who will collect observations. Ensure observers are prepared to address nonadherence.
    - Limit observation periods to no more than 15 minutes.
    - Collect enough observations to detect statistically significant changes in practice.
  - Use an AHHMS to monitor trends in adherence on all shifts and days of the week.^26,163^
    - Collaborate with HCP in the implementation of an AHHMS and empower them to identify ways to improve the system (eg, who to notify when real-time reminders are not accurate or when maintenance is needed).^33,34^
  - Use patient-as-observer methods in areas with limited resources, such as outpatient departments.^38^
  - Use product volume measurement for large-scale planning and benchmarking.
    - Audit the accessibility and functionality of hand hygiene equipment and supplies to ensure hand hygiene is supported by the physical environment of care.^22^
7. **Provide timely and meaningful feedback to enhance a culture of safety.** ^50–52^ (Quality of evidence: MODERATE)
  - Provide feedback in multiple formats (eg, verbal, written) and on multiple occasions (ie, real-time, weekly).^50^
  - Consider debriefing unit managers as soon as possible after each direct covert observation session. This can be done in a manner that preserves the observer’s confidentiality.
  - Provide meaningful data with clear targets linked to actions to improve adherence.^50^
    - Meaningful data may include unit or role-based adherence data rather than overall performance.^164^
    - Real-time displays of hand hygiene adherence may provide incentive for improvement on a shift-by-shift basis.

### Additional approaches to prevent HAIs through hand hygiene during outbreaks

1. **Consider educating HCP using a structured approach (eg, WHO steps) for handwashing or hand sanitizing. Evaluate HCP adherence to technique.** (Quality of evidence: LOW)
2. **For waterborne pathogens in the plumbing of the facility, consider disinfection of sink drains using an EPA-registered disinfectant with claims against biofilms. Consult with state or local public health for assistance in determining appropriate protocols for use and other actions needed to ensure safe supply.** (Quality of evidence: LOW)
3. **For *C. difficile* and norovirus, in addition to contact precautions, encourage hand washing with soap and water after the care of patients with known or suspected infections.** (Quality of evidence: LOW)

### Approaches that should not be considered part of routine hand hygiene

1. **Do not supply individual pocket-sized ABHS dispensers in lieu of minimum thresholds for accessible wall-mounted dispensers.**
2. **Do not refill or “top-off” soap dispensers, moisturizer dispensers, or ABHS dispensers intended for single use.** ^127^
3. **Do not use antimicrobial soaps formulated with triclosan as an active ingredient.** ^67^
4. **Do not routinely double-glove except when specifically recommended for certain job roles or in response to certain high-consequence pathogens**.^141^
  - Certain scrubbed surgical team members must wear double gloves because of the risk for glove perforations that may contaminate sterility or expose the HCP to infectious materials.
  - Anesthesia personnel may wear double gloves during airway management.
  - Personnel compounding medications according to the *US Pharmacopeia* 797 may be required to wear double gloves.
5. **Do not routinely disinfect gloves during care except when specifically recommended in response to certain high-consequence pathogens.**
6. **Do not remove access to ABHS for HCP responding to organisms that are anticipated to be less susceptible to biocides (eg, *C. difficile*, norovirus).** ^11^
7. **Do not attempt to remediate potential biofilms in sink drains with disinfectants lacking EPA registration for this use.**

### Unresolved issues

1. **HCP use of alcohol-impregnated hand wipes is unresolved due to the lack of noninferiority data.^94^**

## Section 5. Performance measures

### Internal reporting

Hand hygiene adherence measurement is not standardized in the United States and will depend on the methods used by the facility and its goal for monitoring. It is intended to support internal quality improvement through measurement, feedback, and longitudinal assessment of interventions at individual facilities or clusters of facilities in the same health system. A list of performance measures for internal reporting is provided in Table 6.

**Table 6.** Metrics for Reporting Adherence to Hand Hygiene

Measurement	Numerator	Denominator	Stratification	Metric
Direct covert observations^a^	No. of adherent hand hygiene opportunities performed	No. of total opportunities	Unit HCP role	(Adherent HHOs)/(Total HHOs) ×100
AHHMS	Approximate no. of hand hygiene actions detected by sensors	Approximate no. of hand hygiene opportunities detected by sensors	Unit HCP role Individual	(Approximate hand hygiene actions)/(approximate HHOs) ×100^a^
Patient as observer	No. of patient reporting adherence	Total number of observations submitted by patients	Service area and/or HCP role	(No. reporting adherence observations)/(Total observations) ×100
Product volume	Volume of hand hygiene product used (eg, alcohol-based hand rub or liquid soap) for a specified period in a specified area	1,000 patient days during specified period in specified area, or number of patient visits for outpatient areas or emergency departments^185^	Unit Service area No stratification (ie, facility-wide)	Volume (mL) per 1,000 patient days or per patient visit
Audits of hand hygiene supplies	No. of hand hygiene stations with defects (eg, lack of adequate supplies or not functioning as intended)	No. of hand hygiene stations assessed	Unit Service area	(No. of hand hygiene stations without defects)/(No. of hand hygiene stations assessed) ×100

a Direct overt observation should not be used to calculate adherence.

### External reporting

There continues to be no requirement in the United States for external reporting of adherence to hand hygiene. Because the credibility of observational methods has yet to be established, any publicly reported hand hygiene metric will suffer from distrust of the data due to misaligned incentives.^1^ When on site, the Centers for Medicaid and Medicare and accrediting organizations [eg, The Joint Commission or Det Norske Veritas (DNV)] evaluate several aspects of hand hygiene programs, including accessibility of supplies, initiatives to improve HCP adherence, measurement methods, and adherence to state-specific administrative code.

## Section 6. Implementation strategies

Leadership at all levels plays a role in hand hygiene improvement, and accountability begins with the chief executive officer and other senior leaders who provide the imperative for HAI prevention, thereby making it an organizational priority. Senior leadership is accountable for providing adequate resources, including necessary personnel, equipment, and assistance when escalating situations of continued nonadherence. Directing interventions toward unit-based managers and department leaders to improve team functioning and supporting their personnel have been reported to be more effective in improving hand hygiene adherence than interventions aimed toward individuals.^165^ Hand hygiene champions, whether informal leaders or formally appointed HCP, have been associated with improved hand hygiene when they provide effective, collegial communication to frontline HCP.^166^

In general, studies examining the association between hand hygiene improvement programs and increases in hand hygiene adherence (and/or decreases in healthcare-associated infections) do not meet the quality standards required of meta-analytic reviews. A Cochrane review published in 2017 included 26 studies of various combinations of interventions, but strategies for hand hygiene improvement were categorized as having low levels of certainty.^170^ Several elements of the implementation strategies are unchanged from those provided in 2014.^1^

### Engage

1. Develop a multidisciplinary team that includes representatives from administrative leadership as well as unit and department managers and unit-level and department-level champions.
  - Align hand hygiene goals with the organizational mission and vision for high-quality patient care.
  - Assure that institutional leadership is aware and supportive of hand hygiene improvement strategies and supports these efforts with adequate resources.
  - When implementing improvement programs, secure the active commitment of unit and department-based leadership. Set targets in collaboration with leaders and teams.^48,171^
    1. Ensure that unit and department managers hold the HCP they supervise accountable for hand hygiene performance.^171^
2. Utilize peer networking to encourage persistent salience of hand hygiene.^1^
  - Consider rewards or recognition for wards modeling good hand hygiene behaviors or improvement. Qualitative studies suggest that role modeling, particularly that of physicians, is important yet underappreciated.^166,172^
3. Identify barriers and facilitators to hand hygiene adherence specific to the unit or institution. Facilitators of adherence may be as simple as having a place to set items prior to entering the patient environment. This information is then used to create interventions specific to their needs.^173,174^
4. Consider enthusiastically inviting patients to take an active role in reminding HCP to perform hand hygiene
  - Ensure HCP respond to patient requests for them to perform hand hygiene in a positive manner.^175^ Consider providing a brief script (ie, “Thank you for reminding me.”)
  - Utilize patient education materials on the CDC website.^176^

### Educate

1. Educate HCP and assure knowledge and skill on the following items:
  - The importance of hand hygiene in reducing the risk for HAI
  - HHOs using WHO Five Moments or CDC indications
  - Fingernail and hand condition, primary prevention of dermatitis
  - Facility-specific policies regarding jewelry
  - Delineation of the patient zone, particularly when patients are housed in bays or crowded areas
  - Technique, ensuring coverage of all hand surfaces, duration of hand rubbing or washing
  - Use of hand-care products that are compatible with hand hygiene products specific to the area in which HCP work
  - Use of gloves in a manner that reduces hand or environmental contamination.
2. Use interactive methods to educate HCP about technique for hand sanitizing, handwashing, and doffing of gloves.
3. Use short, frequent educational interventions to continually build HCP knowledge and practice of hand hygiene.
  - Assess HCP knowledge of hand hygiene with written tests or quizzes.
  - Assess HCP skill in hand hygiene and use of gloves by return demonstration.
4. Use principles of adult education to encourage participation and ongoing learning.

### Execute

1. Provide access to ABHS within the workflow of HCP.
2. Implement a multimodal (ie, bundled) hand hygiene improvement program. Accessibility and visibility of dispensers and supplies may be the most important bundle element.^105,108,177^ A culture of safety that penetrates to the individual level (ie, psychological safety) has also been associated with improved hand hygiene.^178^ Real-time verbal or electronic reminders to perform hand hygiene are likely more effective than signage.^179^ Interventions must be ongoing to maintain behavior change and improved adherence.^180^
3. Focus on targeted behavior change. Posters, if used, should be motivational in nature rather than simply conveying information. Emphasize the protective nature of hand hygiene and altruism.^181^

### Evaluate

1. Measure hand hygiene adherence performance. A combination of approaches may be most appropriate (see Section 2).
2. Measurement may need to be adjusted for facility-specific needs. Use or build upon existing tools:
  - WHO observation forms are available online.^182^
  - A variety of other forms are available for free in The Joint Commission’s hand hygiene monograph.^164^
  - The Joint Commission Center for Transforming Healthcare’s Targeted Solutions Tool for Hand Hygiene are available free for organizations accredited by The Joint Commission.^183^
  - Several iOS and Android applications, including the iScrub application, are available to assist with direct observation.^184^
3. Provide meaningful feedback on hand hygiene performance with clear targets connected with an action plan in place for improving adherence.
  - Feedback of hand hygiene adherence rates has long been recognized as an important component of multimodal hand hygiene improvement program, although the independent impact of feedback apart from other bundled hand hygiene interventions is not known.
  - Feedback may be most effective when provided more than once, when both verbal and written feedback are provided, and when a superior or colleague is responsible for the audit and feedback.^50^
  - Providing overall hand hygiene adherence rates for a facility may not be as effective as unit based or role-based reports at identifying problem areas and planning focused training efforts.
  - Hand hygiene data may be displayed on dashboards that provide the most recent or cumulative hand hygiene adherence rates compared with a target rate. Statistical process control charts can be used to show data trends over time and whether changes in rates are due to specific interventions or normal variation. Some automated monitoring systems can give real-time displays of hand hygiene adherence on the unit, providing some incentive for improvement on a shift-by-shift basis.
  - Use feedback to engage HCP in identifying problems at individual hospital or unit level and use data to tailor ongoing interventions.
  - If individually identified hand hygiene adherence rates are used, consider providing feedback privately versus in a public staff setting.
  - Some facilities report hand hygiene adherence data in conjunction with hospital-associated infection rates. Although the association between hand hygiene and HAI reductions has been reported in the literature, the association may not be evident in individual unit or facility data due to confounding factors (eg, environmental cleanliness and small sample sizes).
  - Use monitoring data to inform action plans at the most specific level possible (administrative area, service line, unit, or even individual) and follow through on improving these process measures as a step towards improving hand hygiene overall.

Hand hygiene programs should strive to create a culture of safety in which all HCP collaborate to protect patients or residents. Interprofessional dialogue and safe spaces for learning about hand hygiene provide motivation and engagement of HCP.^167^ Strategies for implementation of multimodal hand hygiene improvement programs, including system and/or infrastructure change (eg, availability of alcohol-based hand rubs), education, evaluation and feedback, reminders (eg, posters), and institutional safety climate (eg, administrative support), have been endorsed and detailed by the WHO in the 2009 publication entitled *A Guide to the Implementation of the WHO Multimodal Hand Hygiene Improvement Strategy*.^11,168^ Resources are available on the CDC Hand Hygiene in Healthcare Settings webpage and from other organizations such as The Institute for Healthcare Improvement’s “How to” guide^169^ and The Joint Commission Center for Transforming Healthcare Targeted Solutions Tool (or TST) for Hand Hygiene.^171^
